# Effect of TX-100 pretreatment on carbon paste electrode for selective sensing of dopamine in presence of paracetamol

**DOI:** 10.1038/s41598-022-24387-z

**Published:** 2022-11-24

**Authors:** J. K. Shashi Kumara, B. E. Kumara Swamy, G. K. Jayaprakash, S. C. Sharma, Roberto Flores.-Moreno, Kaustubha Mohanty, S. A. Hariprasad

**Affiliations:** 1 Department of P.G. Studies and Research in Industrial Chemistry, JnanaSahyadri, Kuvempu University, Shankaraghatta, Shivamogga, Karnataka 577451 India; 2 Department of Chemistry, Nitte Meenakshi Institute of Technology, Bangalore, Karnataka 560064 India; 3 National Assessment and Accreditation Council (Work Carried Out As Honorary Professor), Jain University, Bangalore, Karnataka 560 069 India; 4 School of Energy Science and Engineering, Indian Institute of Technology Guwahati, Guwahati, 781039 India; 5 Departamento de Química, Centro Universitario de Ciencias Exactas e Ingenierías, Universidad Guadalajara, Blvd. Marcelino Garcı´a Barraga´N 1421, C.P. 44430 Guadalajara, Jal. Mexico; 6 Department of Chemical Engineering, Indian lnstitute of Technology Guwahati, Guwahati, 781039 India; 7 Jain University, Bangalore, Karnataka 560 069 India

**Keywords:** Biochemistry, Chemistry, Engineering

## Abstract

Dopamine (DA) is one of the chief neurotransmitters present in the central nervous system of mammals. Therefore detection of DA in presence of various analytes like paracetamol has great importance. In the current work, we are proposing that Triton X-100 (TX-100) pretreated carbon paste electrode (CPE) can be useful to detect the DA selectively in presence of PA. After the pretreatment CPE can detect DA in presence of PA effectively. Cyclic voltammetry was employed to observe the amplified electron transfer reaction between the modified CPE and DA. To understand electron transfer regioselectivity at the TX-100 pretreated CPE, a dual descriptor was used. The prepared electrode showed satisfactory stability when kept under ambient conditions. The proposed approach also showed excellent analytical applicability to identify DA and PA in commercial formulations. The scope of the work is limited to detecting DA in presence of PA. We will consider the other interferes for future works.

## Introduction

Now-day researchers are more concentrating on carbon paste electrodes (CPEs) to utilize them as working electrodes in electrochemical sensors applications. CPEs offer several appealing properties, including surface repeatability, stability, and renewability, making them appealing substances for working electrodes^1–3^. Due to their inexpensive cost relative to other materials, CPEs are becoming more widely used in areas such as pharmaceutical, biological, and environmental analysis^4,5^.

Physical or chemical modifications can improve CPE's sensing properties like selectivity, adsorption capacity, and sensitivity^6,7^. The goal of modifying a CPE matrix is to create an efficient electrode with sensing properties that will be useful for users. In general, after modifications, electrocatalytic activities at the CPE interface will improve, which may be due to the increased surface area which in turn gives better current sensitivity for electron transfer reactions. CPE can be chemically modified using conductive substrates modified with electroactive thin films, monolayers, or thick coatings^8,9^.

The electrochemical modifications may include the use of different types of surfactants to modify the CPE. Clay minerals treated with surfactants have excellent sorption capabilities for both organic and inorganic substances in aqueous solutions^10,11^. In terms of electrode modification, T-octylphenoxypolyethoxyethanol (Triton X-100 (TX100)) is one of the best nonionic surfactants^12–16^. The properties of hybrid materials (carbon paste + TX-100) are highly promising. TX-100 modified carbon hybrid materials are previously reported to use as electrodes. TX-100 adsorbed electrode alters the electrode–electrolyte interface, making it easier to create poly (m-toluidine) films with higher current densities^16^. TX-100 modified CPE was prepared by surface immobilization technique by B.N. Chandrashekar et al. and this method exhibit good sensitivity, but TX-100 pretreatment is more applicable than this method^17^. According to the literature, modification of electrodes using TX-100 has several works but the pre-treatment method good one for analysis of biomolecules proved by the present work^18,19^.

The modifications of electrodes can be done by methods like mechanical grinding, self-assembled layers, covalently bonded electrolyzers, surfactant immobilization, etc. The electrochemical pre-treatment process increases the electron transfer compared to others. To use carbon surface effectively one must take care of the degree of activation^20^. The modification of CPEs is achieved simply by pre-treatment of TX-100 modifier with the carbon paste which is enhancing the sensitivity and selectivity of the electrochemical analysis technique.

Because of its simplicity, sensitivity, efficiency, and low cost, electrochemical pretreatment was chosen for this study. Pretreatment is considered environmentally benign since it uses less dangerous chemicals than other time-consuming and sophisticated modification materials. The electrochemical pretreatment procedure results in transducers that produce signals that are undistorted, well-defined, and repeatable. By creating charge-carrying functional groups, the pretreatment of the CPE enhances the selectivity and sensitivity of the sensors for detecting selected electroactive components. In the present work, we study the effect of TX-100 pretreatment on CPE, and it is used in applications for DA and PA sensors with performing the detection of DA in the presence of PA because the usage of PA protects dopaminergic neurons against oxidative stress damage produced by acute exposure to increased amounts of DA, according to in vitro studies^21^ and it shows outstanding stability, sensitivity, and selectivity for biomedical applications.

## Experimental section

### Reagents and chemicals

Paracetamol (PA) was obtained from Merck, sodium hydroxide (NaOH), and dopamine hydrochlorides (DA) from nice chemicals. The graphite powder (LobaChemie), Silicone oil and TX-100 are obtained from Himedia.

### Apparatus

The TX-100 atomic model has created with Sinapsis software (Version: Sinapsis.XIII Weblink: http://sinapsis.sourceforge.net Acess date: accessed July 20, 2020)^22^. The experiments were done using the potentiostat model CHI-660c (CH Instrument-660 electrochemical workstation) and the generated data was transferred to origin 6.1. Bare CPE (BCPE), NaOH pretreated modified CPE (NaOH-PT-MCPE), and TX-100 pretreated modified CPE (TX-100PT-MCPE) are used as working electrodes, saturated calomel as the reference electrode, and a platinum electrode as a counter electrode.

### Preparation of BCPE

The BCPE was prepared by hand mixing 70% graphite powder with 30% silicon oil in an agate mortar for approximately 30 min to produce a homogenous carbon paste. The paste was packed into the homemade cavity of a Teflon tube and polished on a paper napkin to obtain a uniform surface**.**

### Pretreatment of CPE with TX-100

**Figure 1:**
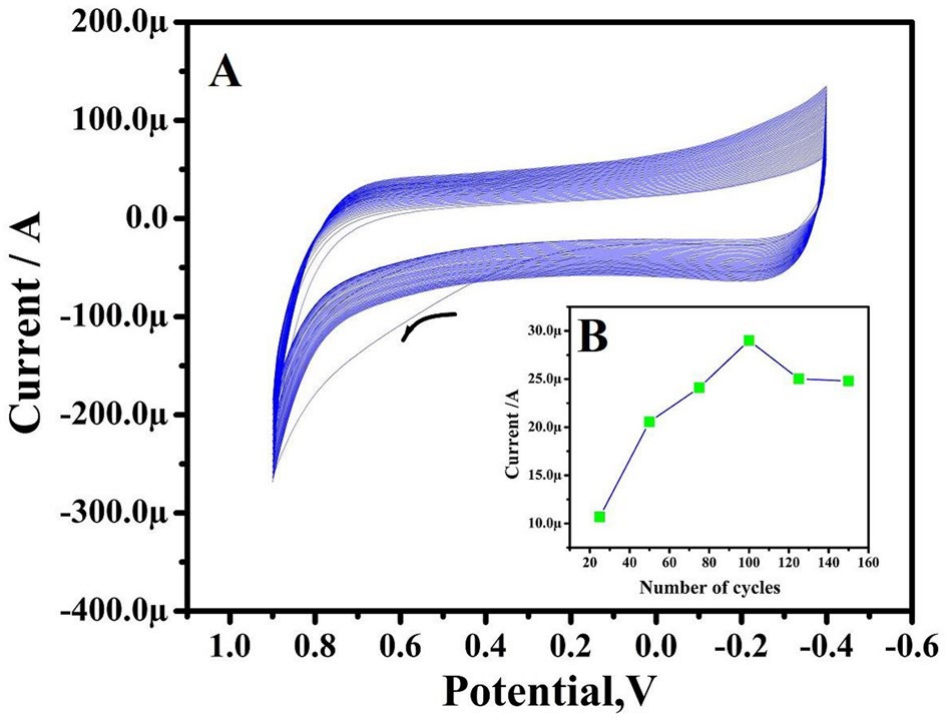
(**A**) CVs for electrodeposition of TX-100 on a BCPE in 0.1 M NaOH by 100 cycles. Sweep rate: 1 Vs^−1^. (**B**)The graph of anodic peak current versus several cycles for potassium ferrocyanide at a sweep rate of 50 mV/s.

The pretreatment process was carried out using cyclic voltammetry (CV), potential cycles were between − 0.4 and 0.9 V at a scan rate (ν) of 1 V/s for 100 cycles as depicted in Fig. 1A. With an increase in the cycle number voltammogram, currents decreased gradually which indicates that during the pretreatment process TX-100 is adsorbing on the electrode surface. To eliminate contaminants electrode was dipped in deionized water. The electrochemical response of TX-100PT-MCPE with different cycles is measured by using potassium ferrocyanide, K_4_(Fe (CN)_6_) at a sweep rate of 50 mV/s is depicted in Fig. 1B. TX-100PT-MCPE prepared by 100 cycles showed better sensing capability hence it is considered for further analysis.

### Computational analysis

**Figure 2:**
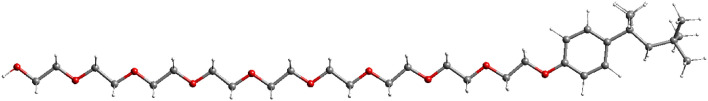
Atomistic model of Triton X-100 (H = white, C = grey, and O = red).

The TX-100 atomic model was created with Sinapsis software^22^ as shown in Fig. 2 and geometry optimization was done with deMon2k software^23^ using PBE exchange - correlation functions^24,25^ and TZVP basis sets^26^, employing density functional theory (DFT) as described in our previous articles. Sinapsis software was used to plot the dual descriptor results.

## Results and discussion

### The PTTMCPE electrochemical response towards potassium ferrocyanide

**Figure 3:**
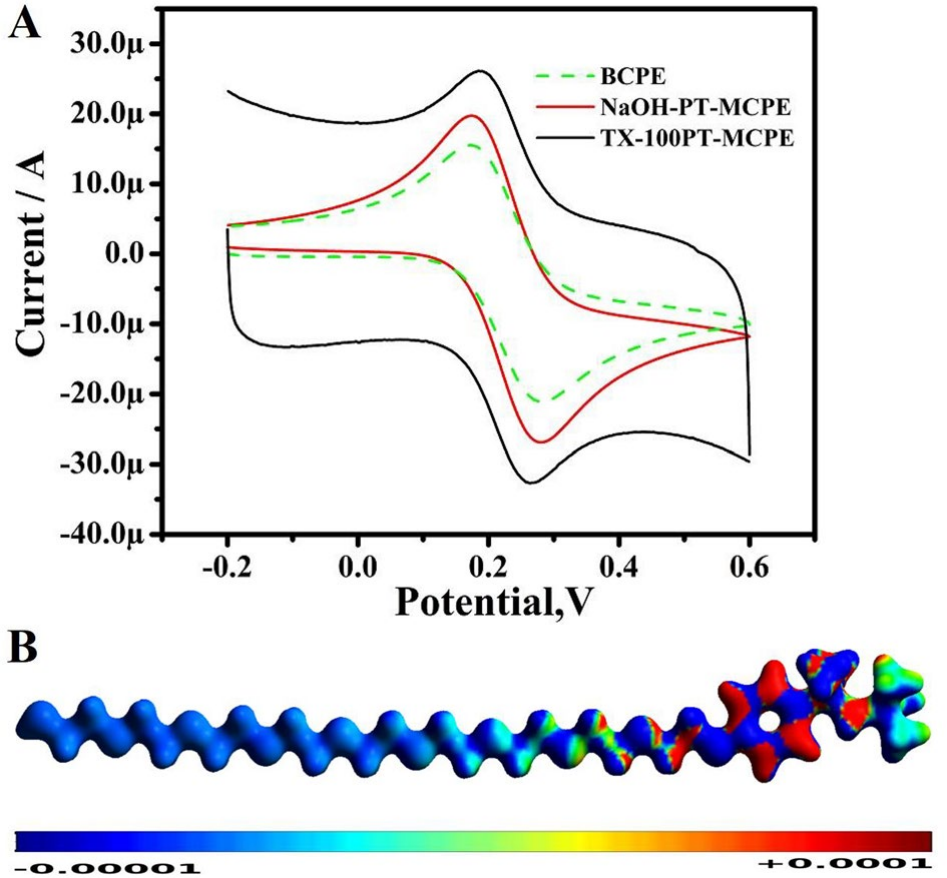
(**A**) CVs of 1 mM potassium ferrocyanide in 1 M KCl solution at a speed rate of 50 mV/s. (**B**) Dual descriptor result of TX-100 [ISO = 0.07, grid = 0.3].

The cyclic voltammetric (CV) response of K_4_(Fe(CN)_6_ at TX-100-PTCPE is shown in Fig. 3A. The BCPEs had poor peak current and high redox peak potential differences, but the MCPEs had much-improved redox peak current signals and smaller redox peak potential discrepancies. Randle’s Sevick's equation^27^ was used to calculate the total active surface area. The active surface area for electrodes (BCPE, NaOH-PT-MCPE, and TX-100PT-MCPE) is shown in Table 1.

**Table 1:** The electrochemical performance of the electrodes.

Electrodes	Ipa (A)	∆E (V) [at *ν* = 0.05 V]	Area (cm^2^)
BCPE	2.12 × 10^–05^	0.110	0.0321
Pretreated NaOH MCPE	2.70 × 10^–05^	0.103	0.0372
PTTMCPE	3.26 × 10^–05^	0.050	0.0412

The heterogeneous rate constant (k^0^) values for K_4_(Fe (CN)_6_ are calculated from the ΔEp values (> 10 mV) using Eq. (1). The k^0^ values are listed in Table 2.

1 $$\Delta {\text{Ep}} = 201.39 {\rm log}(\upsilon /{\text{k}}^{0} ) - 301.78$$

**Table 2:** The rate constant values for potassium ferrocyanide at different electrodes.

υ/mV/s	ΔEp/mV		k°(s^−1^)			
	BCPE	Pretreated NaOH MCPE	PTT MCPE	BCPE	Pretreated NaOH MCPE	PTT MCPE
50	110	103	50	0.895798	0.488696	0.451108
100	131	130	82	0.709635	0.717796	1.242637
150	141	140	87	0.949449	0.960367	1.760387
200	153	150	97	1.103633	1.142144	2.093592
250	159	158	104	1.288076	1.302888	2.415703

TX-100 redox sites are found using ADPT-based dual descriptor calculations. The dual descriptors or dual Fukui functions are obtained using Fukui functions. Fukui functions can be defined according to Eq. (2)^26^. Previously we have used the Fukui functions to analyze surfactants^28,29^.

2 $${f}^{\pm }\left({\varvec{r}}\right)\equiv \underset{\Delta N\to {0}^{\pm }}{\mathrm{lim}}\frac{{\rho }_{N+\Delta N}\left({\varvec{r}}\right)-{\rho }_{N}\left({\varvec{r}}\right)}{\Delta N}$$

where N is the number of electrons in the system, *ρ*(**r**) is the electron density, + and − signs correspond to the addition or remotion of electrons, respectively. The dual descriptor (${f}_{D}$) is plotted using Eq. (3).

3 $${f}_{D} = {f}^{+} - {f}^{-}$$

The dual descriptor charts for TX-100 are shown in Fig. 3B^22^. The blue colour regions in our dual descriptor maps indicate TX-100 oxidation sites, whereas the red colour regions represent TX-100 reduction sites. TX-100 is oxidized via the tail due to the presence of oxygen atoms with lone pair electrons, and it is reduced via the head phenyl ring.

### The electrochemical response for DA at PTTMCPE

**Figure 4:**
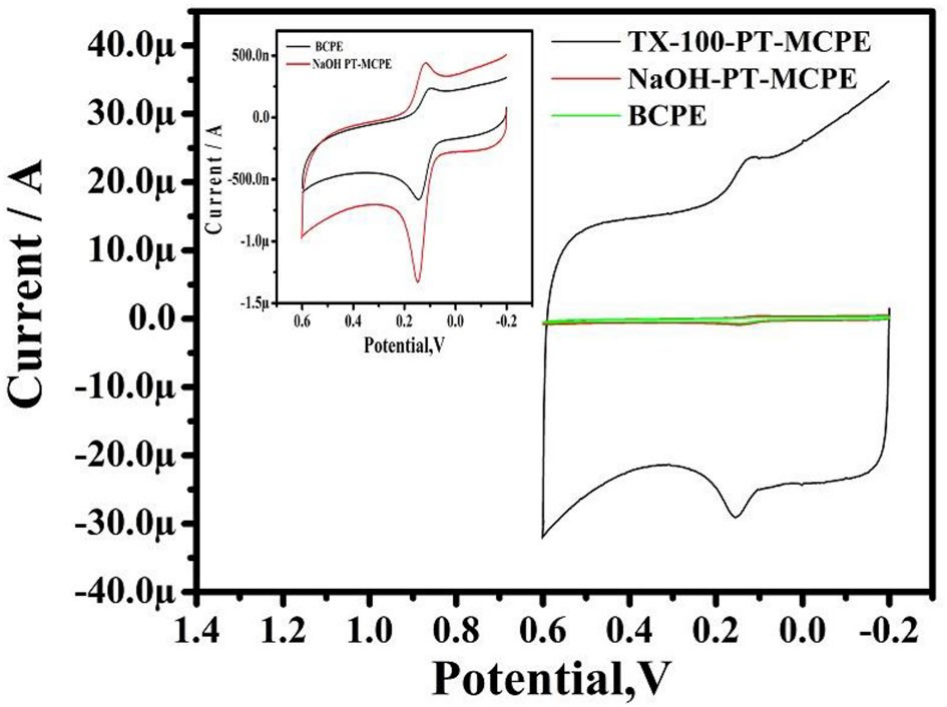
CVs for 10 µM DA in 0.2 M PBS solution of pH 7.4 at pretreated NaOH and TX-100-PT-MCPE at the sweep rate of 100 mV/s. Inset: compares the BCPE and NaOH pretreatment.

The voltammetric response of DA (10 µM) at the BCPE, NaOH-PTMCPE, and TX-100PT-MCPE was displayed in Fig. 4. The peak potential difference (ΔEp) was 58 mV at BCPE. However, ΔEp values are 28 mV and 25 mV for NaOH-PT-MCPE and TX-100-PTMCPE respectively. This indicates that pretreated electrodes are good for sensing applications.

### Electrochemical investigation of DA with the different parameters at TX-100-PT-MCPE

**Figure 5:**
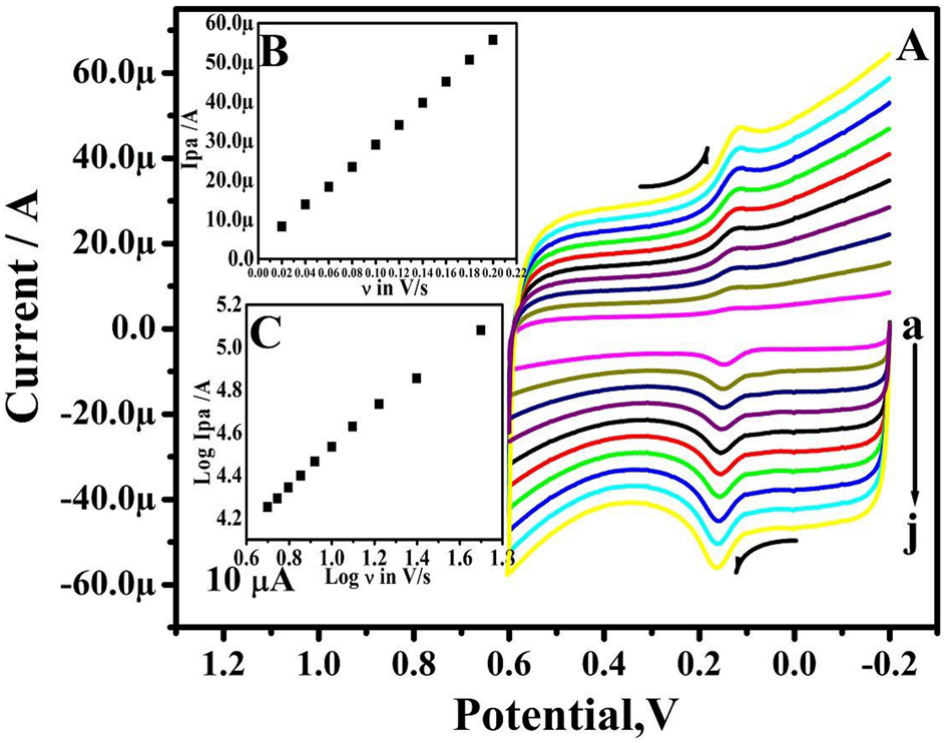
(**A**) CVs of 10 µM DA in 0.2 M PBS solution of pH 7.4 at TX-100-PT-MCPE at different sweep rates. (a–j; 20–200 mV/s). (**B**) Graph of peak current versus sweep rate. (**C**) Graph of the log of peak current versus the log of sweep rate.

As shown in Fig. 5A, the effect of change in the ν for 10 µM DA in 0.2 M PBS was studied by CV at TX-100PT-MCPE. The TX-100PT-MCPE demonstrates that as the ν increases the redox peak current steadily increases. The plot of anodic peak current (Ipa) vs ν verifies the type of electrode process (Inset of Fig. 5B). The correlation coefficient was originally set at R^2^ = 0.9997, however, Fig. 5C depicts a plot of log Ipa vs log ν with 0.8362 slopes. Therefore, the mass transfer process is adsorption controlled^30^.

**Figure 6:**
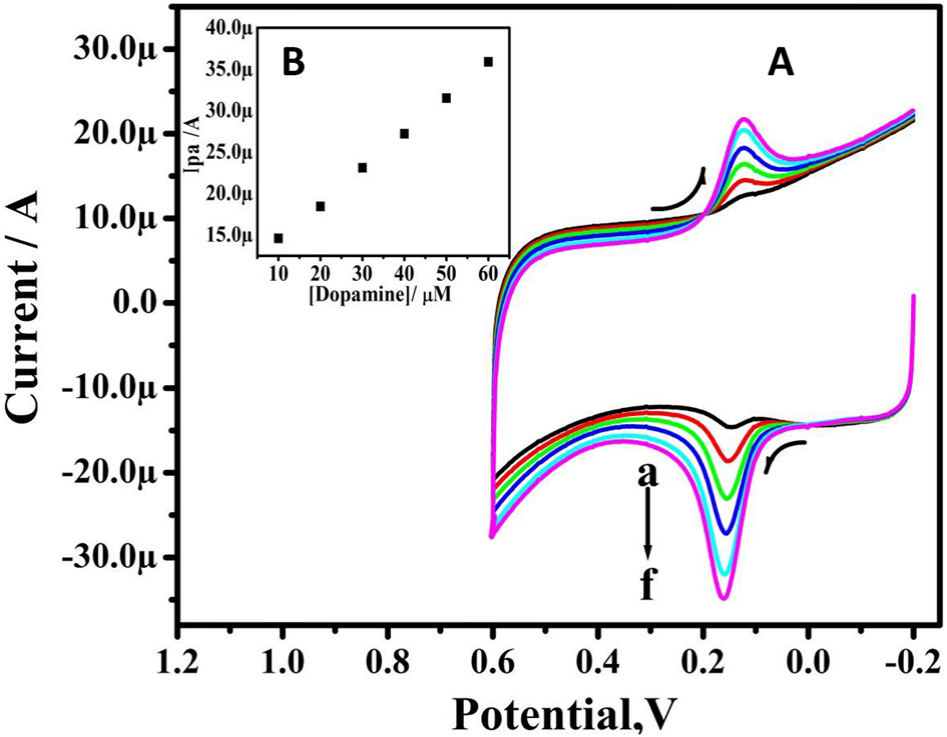
(**A**) CVs of 10 µM DA in 0.2 M PBS solution of pH 7.4 at TX-100-PT-MCPE at a sweep rate of 50 mV/s with different concentrations (a–f: 10–60 μM). (**B**) Graph of anodic peak current versus concentration of DA.

Figure 6A displays the CV response of DA at TX-100-PT-MCPE at different concentrations of 10–60 μM using 0.2 M PBS (pH 7.4) with a ν 100 mV/s. With a rise in concentration, the corresponding anodic peak current (Ipa) of DA enhances. Figure 6B shows the curve of Ipa vs DA concentration. It has high linearity with regression equations are Ipa(μA) = 42 (μM) + 0.105 (R^2^ = 0.9981) and the limit of detection (LOD) and limit of quantification (LOQ) are calculated according to Eqs. (4, 5)^30^. Where S is the standard deviation and M is the slope obtained from the calibration plot:

4 $$\mathrm{LOD }= 3\mathrm{ S}/\mathrm{M}$$

5 $$\mathrm{LOQ }= 10\mathrm{ S}/\mathrm{M}$$

The comparative analytical performance electrode for DA was presented in Table 3^31–47^ and LOQ was found to be 4.08 μM.

**Table 3:** Comparative analytical performance electrode for DA.

Sl. no.	Electrodes	Linear range (µM)	LOD (µM)	Method	References
01	Au/Gr-Au	0.03–30	30	SW	^31^
02	Pt–Au hybrid	103–165	24	CV	^32^
03	CTAB/CPE	0–130	11.0	DPV	^33^
04	Fc-MCPE	120–11,000	9.4	CV	^34^
05	Poly (Sudan III)/MCPE	10–60	9.3	CV	^35^
06	SWCNT/GCE	–	7.0	DPV	^36^
07	Metallothioneins self-assembled gold electrode	0.08–0.2	6.0	CV	^37^
08	LDH/CILE	10–1100	5.0	DPV	^38^
09	Ag-reduced GO/GCE	10–800	5.4	LSV	^39^
10	Poly-VA/MWCNT/GCE	5–120	4.5	CV	^40^
11	Ag/Ag2S-CNT-Nafion	250–2000	4.7	DPV	^41^
12	BPVCM-e/MWCNT/GCE	5–5000	2.25	CV	^42^
13	Poly (amido black) MCPE	10–60	2.03	CV	^43^
14	CTAB-GO/MWNT	5.0–500	1.5	DPV	^44^
15	TX-100-PT-MCPE	10–60	1.22	CV	Present work
16	SiTi/AuNP/CPE	20–180	0.57	CV	^45^
17	Ni-Zr/MSN/GCE	0.3–100	0.13	CV	^46^
18	PSi NPs modified GCE	0.5–333.3	0.032	CV	^47^

**Figure 7:**
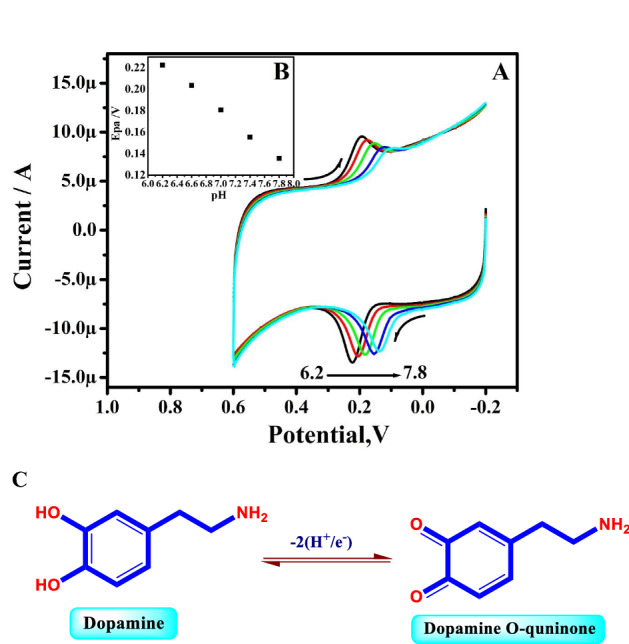
(**A**) CVs of 10 µM DA for different pH (6.2–7.8 pH) at TX-100-PT-MCPE. (**B**) Graph of Epa versus different pH. (**C**) Redox mechanism dopamine.

The CVs for DA obtained by the TX-100-PT-MCPE at ν of 100 mV/s in PBS of various pH from 6.2 to 7.8 for DA are shown in Fig. 7A. The Ipa of DA changed to a less positive potential as the pH increased. A graph of Epa vs different pH levels was used to create the potential diagram (Fig. 7B). With a slope of 55 mV/pH, the obtained graphs exhibit acceptable linearity with regression equations are E_pDA_ (mV) =  − 55 pH + 56 (R^2^ = 0.9989). The calculated slope is extremely near to the Nernstian value of 59 mV, as shown in the graphs. Therefore, an equal number of protons and electrons are transferred in the reactions (Fig. 7C).

### Electrochemical investigation of PA with different parameters at TX-100PT-MCPE

**Figure 8:**
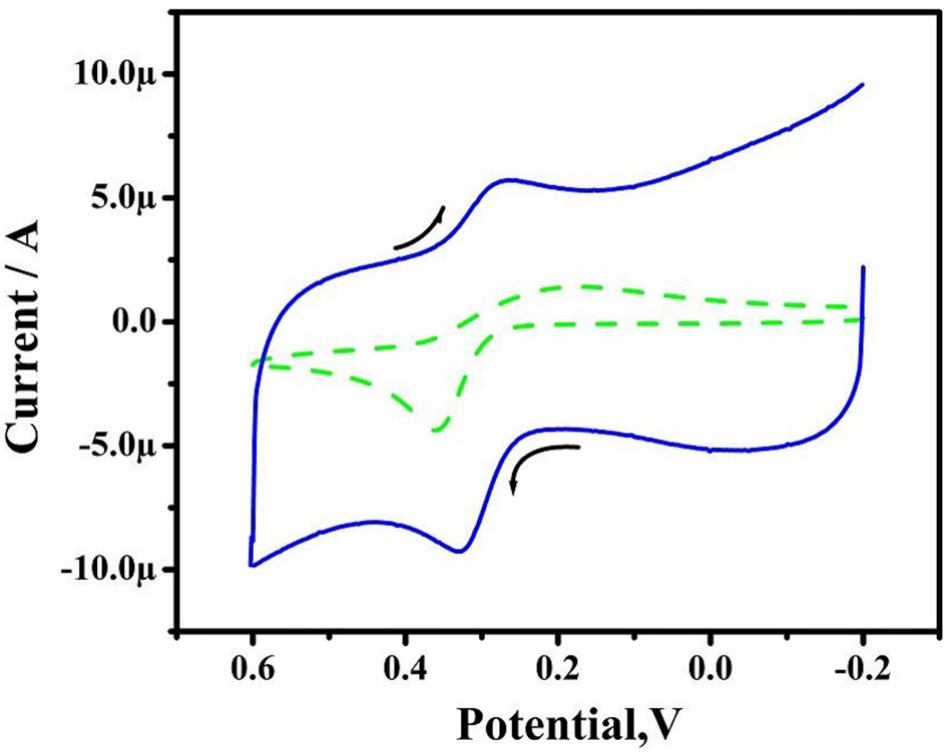
CVs of 10 µM PA in 0.2 M PBS solution of pH 7.4 at BCPE and TX-100-PT-MCPE at the sweep rate of 100 mV/s.

A CV profile of 10 μM PA at BCPE and TX-100PT-MCPE in 0.2 M PBS is shown in Fig. 8 (pH 7.4). The TX-100PT-MCPE shows an increase in redox Ipa and a small shift in potential, but the BCPE, PA shows less current and more widespread peaks. Our findings show that following the change, the electrode performs well and lowers ΔE while increasing Ipa. These findings show that the proposed sensor has a catalytic effect on PA analysis.

**Figure 9:**
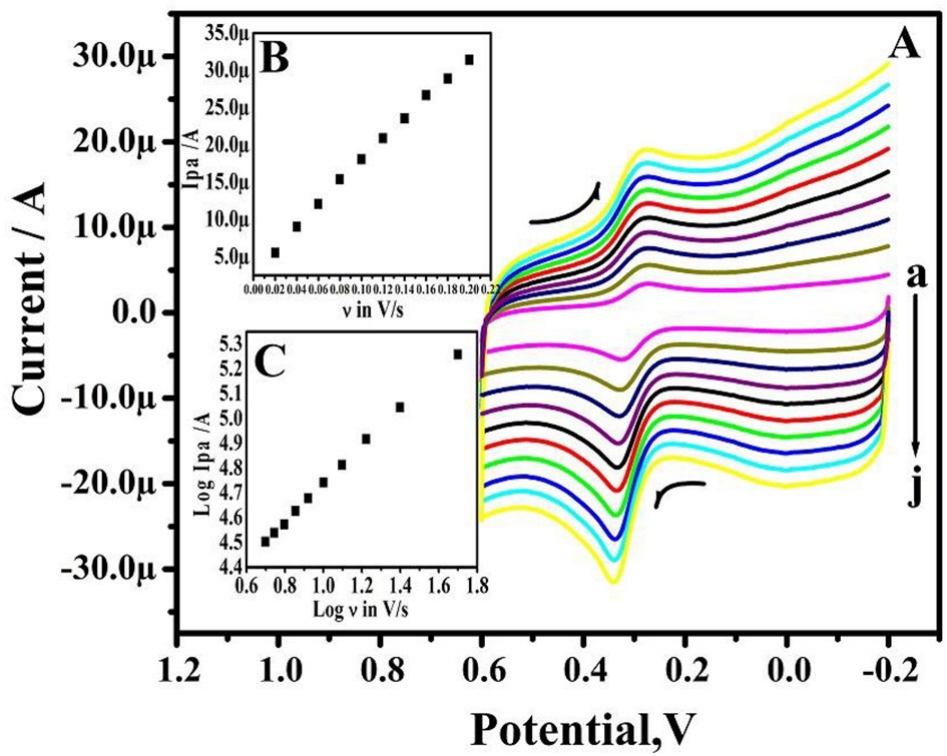
(**A**) Cyclic voltammograms of 10 µM PA in 0.2 M PBS solution of pH 7.4 at TX-100-PT-MCPE at different sweep rates. (a–j; 20–200 mV/s). (**B**) Graph of peak current versus sweep rate. (**C**) Graph of the log of peak current versus the log of sweep rate.

At TX-100-PT-MCPE, the effect of ν on the CV performance of the PA was investigated in PBS (pH 7.4). Ipa increased with a slight positive shift in the peak potential displayed in Fig. 9A increased in the region of 20–200 mV/s. In Fig. 9B,C, the current intensity changes in terms of sweep rates are shown in a graph with good linearity. The findings show that the oxidation–reduction process of PA is influenced by adsorption.

**Figure 10:**
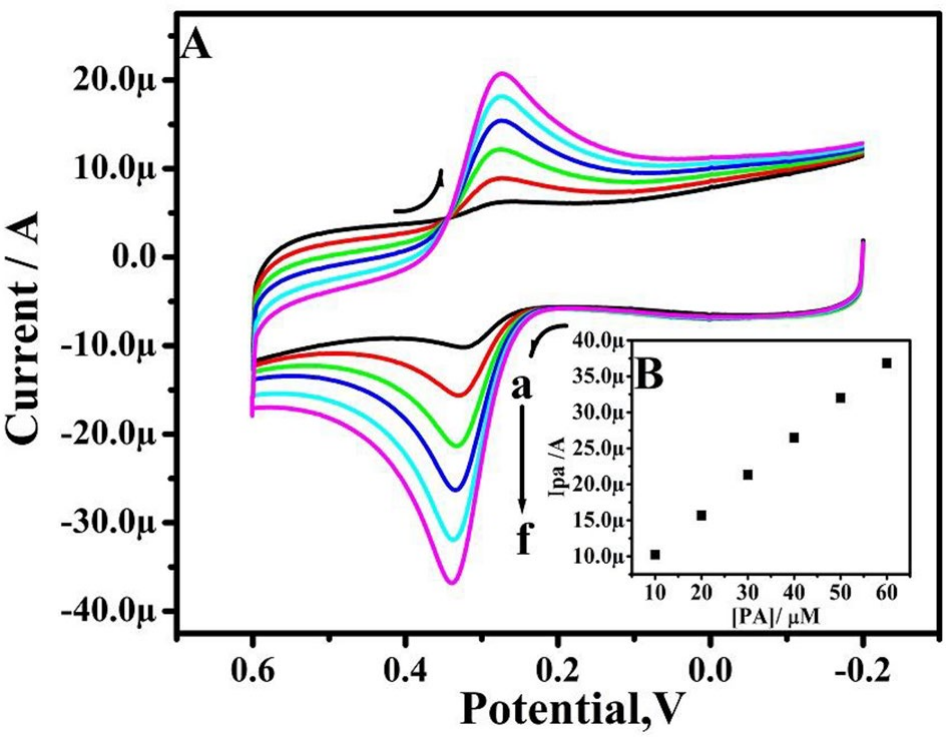
(**A**) CVs of 10 µM PA in 0.2 M PBS solution of pH 7.4 at TX-100-PT-MCPE at a sweep rate of 50 mV/s with different concentrations (a–f: 10–60 μM). (**B**) Graph of anodic peak current versus concentration of PA.

PA CVs at TX-100-PT-MCPE at various concentrations are depicted in Fig. 10A. PA's Ipa hikes with an increase in concentration shown in Fig. 10B. The linearity of the Ipa vs. PA concentration was found high linearity with regression equations are Ipa(μA) = 01 (μM) + 0.15 (R^2^ = 0.99979) and the limit of detection (LOD) and limit of quantification (LOQ) are calculated according to Eqs. (4, 5) and are found to be 5.031 μM and 16.77 μM respectively.

**Figure 11:**
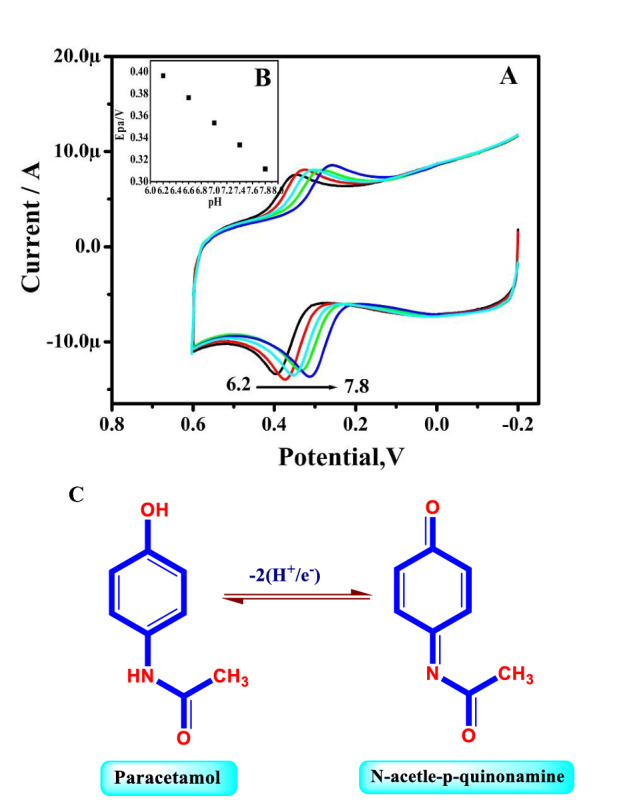
(**A**) Cyclic voltammograms of 10 µM PA for different pH (from a–e; 6.2–7.8 pH) at pretreated TX-100 modified CPE. (**B**) Graph of Epa versus different pH. (**C**) Redox mechanism paracetamol.

The CVs profile for PA, at TX-100-PT-MCPE in 0.2 M PBS range 6.2–7.8 is portrayed in Fig. 11A. The Epa was moved negative with an increase in pH values. Figure 11B portrayed the linear establishment between Epa and pH and provide a slope of 57 mV for PA with regression equations are E_pDA_ (mV) =  − 57 pH + 58 (R^2^ = 0.9997). The resulting slope is close to the Nernstian value. Therefore, an equal number of protons and electrons are transferred in the reactions (Fig. 11C).

### Selectivity and reproducibility study for TX-100-PT-MCPE

**Figure 12:**
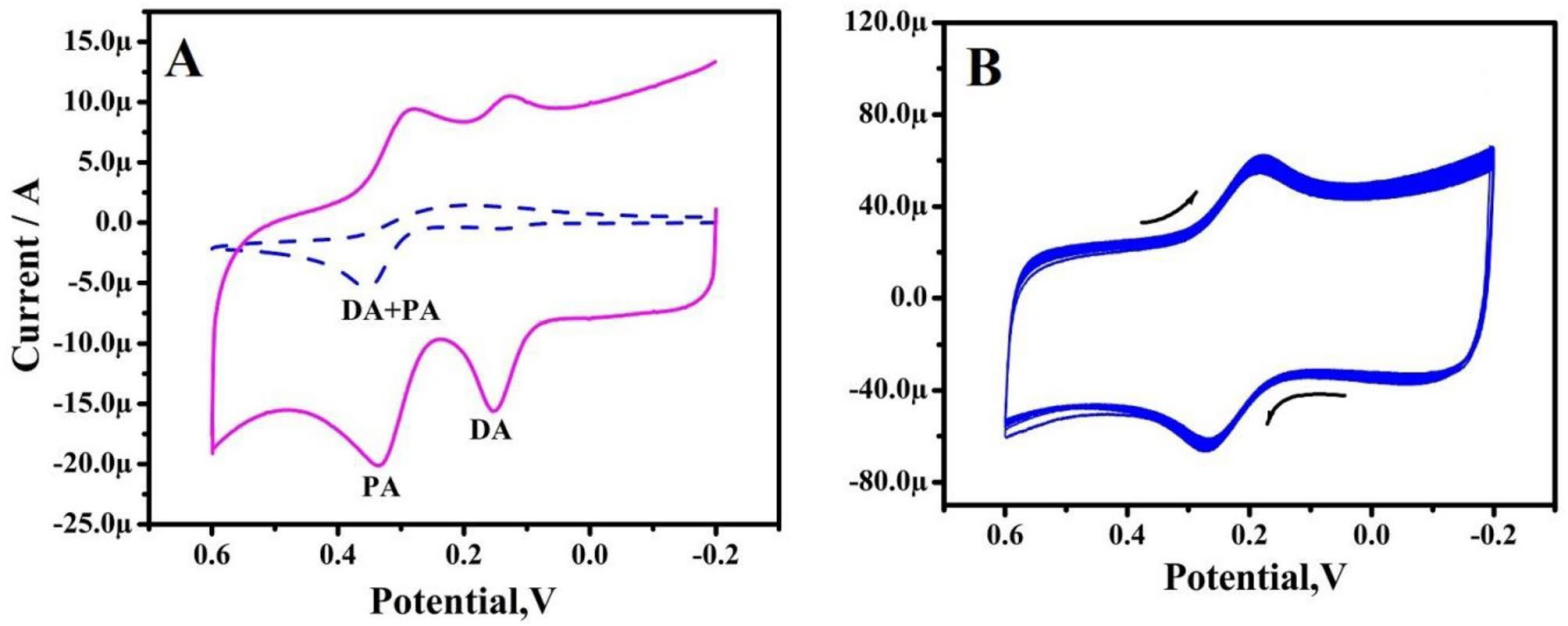
(**A**) CVs for simultaneous determination for 10 µM DA and 10 µM PA at BCPE (dashed line) and TX-100-PT-MCPE (solid line) at a sweep rate of 100 mV/s. (**B**) CVs of 1 mM potassium ferrocyanide in 1 M KCl solution at a speed rate of 50 mV/s for stability analysis for TX-100-PT-MCPE at 50 cycles.

Figure 12A displays the CV responses of 10 µM of DA and 10 µM PA in 0.2 M PBS of both BCPE and TX-100PT-MCPE. The BCPE showed short current signals with poor selectivity indicating that electrode is not selective for simultaneous detection of DA and PA. But after TX-100 pretreatment current signals are enhanced and selectivity was improved (oxidation potential of DA and PA are separated). Therefore, TX-100PT-MCPE can effectively be used to detect DA in presence of PA. The fabrication reproducibility was checked by successively scanning 50 cycle’s depicted in Fig. 12B. The Ip signals retained approximately 90.6% of the original value. The experimental results indicated that the TX-100PT-MCPE possesses a long service life.

### Interference study

**Figure 13:**
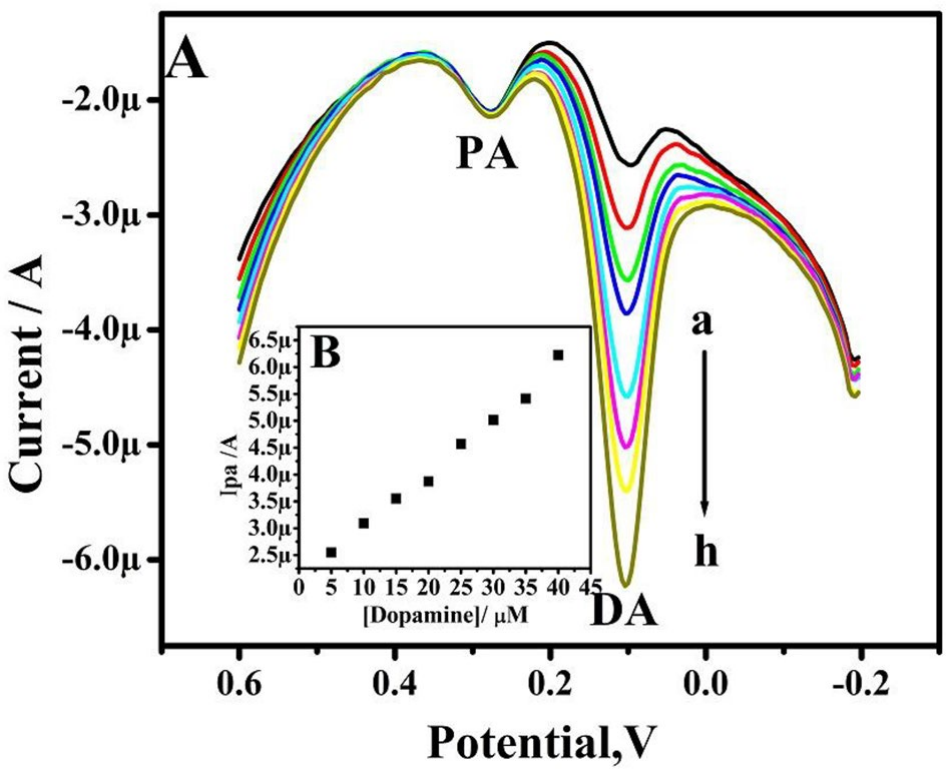
(**A**) Differential pulse voltammograms were obtained for variation of concentration 5–40 µM (a–h) DA, in PBS (pH 7.4), in the presence of 5 µM PA at TX-100-PT-MCPE. (**B**) Graph of anodic peak current versus concentration of DA.

Figure 13A displays the DPVs of DA at 5–40 µM concentration in presence of 5 µM PA. As a result, the Ipa of DA increases with an increase in DA concentration, and the Ipa of PA remains constant. The obtained results are good as shown in the graph of Ipa versus the concentration of DA (Fig. 13B). This indicates that TX-100-PT-MCPE can selectively sense DA in presence of PA.

**Figure 14:**
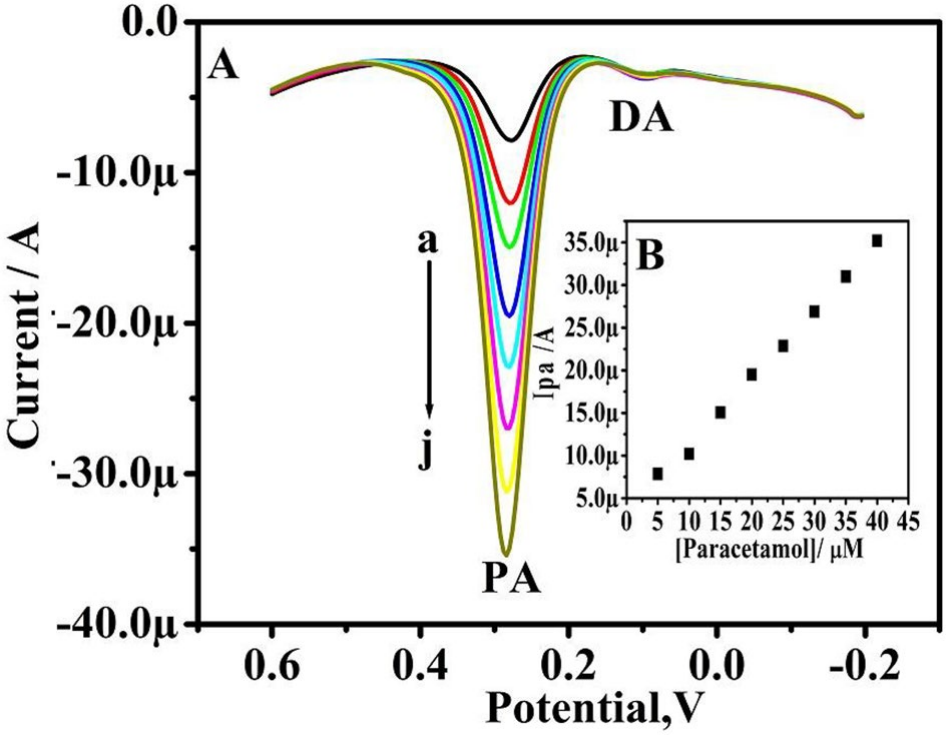
(**A**) Differential pulse voltammograms obtained for variation of concentration 5–40 µM (a–h) PA, in PBS (pH 7.4), in the presence of 5 µM DA at pretreated TX-100 modified CPE. (**B**) Graph of anodic peak current versus concentration of PA.

Figure 14A demonstrates raising the concentration of PA from 5 to 40 µM while maintaining the DA concentration constant (5 µM). The curve of Ipa vs concentration of PA is depicted in Fig. 14B and it is linear. The result confirms that changing the concentration of one analyte does not affect the peak current and peak potential of another analyte; it also demonstrates the modified carbon paste electrode's outstanding selectivity and sensitivity towards DA and PA.

### Real sample analysis

For the examination of DA-containing injectable samples and PA tablet samples by standard addition method using TX-100PT-MCPE. The injection samples and DA injection were utilized after a sufficient dilution with a 0.2 M phosphate buffer. Dolo-500, Micro Labs Ltd provided the PA tablet sample, which had a PA 500 mg concentration. The obtained results are tabulated in Table 4. The recovery was acceptable, indicating that the proposed methods may be utilized to detect DA in injections with a recovery rate of 98.20–99.10 percent and a recovery rate of 98.5–99.45 percent for the PA tablet sample.

**Table 4:** Detection of DA and PA in the real sample (n = 3).

Sample	DA added (µM)	Found (µM)	Recovery (%)
DA injections	10	9.84	98.4 ± 0.976
	20	19.46	98.20 ± 0.876
	30	29.73	99.10 ± 0.135
PA tablet	10	9.85	98.5 ± 0.911
	20	19.69	98.45 ± 0.992
	30	29.28	97.6 ± 0.997

## Conclusion

The pretreatment of the CPE with TX-100 in 0.1 M NaOH solution results in a very stable DA sensor with outstanding sensitivity and selectivity. The TX-100PT-MCPE reduced the overpotential and enhanced the current responsiveness. The pH study reveals that the redox mechanism involves an equal quantity of protons and electrons being transferred. This demonstrated a promising biological use. The prepared electrode showed satisfactory stability when kept under ambient conditions. The proposed approach is also used to identify DA in injectable samples and PA in commercial tablet formulations.
